# A randomized clinical trial of transdermal (gel) versus oral estrogen for endometrial preparation in frozen embryo transfer cycle

**DOI:** 10.1590/1806-9282.20231548

**Published:** 2024-05-20

**Authors:** Mariana Oliva Cassará Carvalho, Sônia Maria Rolim Rosa Lima, Claudia Godman Glina, Leopoldo de Oliveira Tso, Rodrigo Sabato Romano, Sidney Glina, Newton Eduardo Busso, Cristiano Eduardo Busso

**Affiliations:** 1 Santa Casa de Sao Paulo School of Medical Sciences, Department of Obstetrics and Gynecology – São Paulo (SP), Brazil.; 2 Project Alliance of Assisted Fertility Laboratories/BETA – São Paulo (SP), Brazil.

**Keywords:** Embryo transfer, Estrogens, Cutaneous administration, Oral administration, Endometrium

## Abstract

**OBJECTIVE:**

The aim of this study was to compare endometrial thickness with the use of transdermal estrogen (gel) versus oral estrogen (pills) for endometrial preparation in the frozen embryo transfer cycle and serum estrogen concentrations during the preparation cycle, side effects, and chemical and clinical pregnancy rates.

**METHODS:**

This was a prospective, randomized controlled trial of women undergoing endometrial preparation for cryopreserved blastocyst transfer. A total of 88 women were randomized, of which 82 completed the study protocol. Of this group, 44 received 6 mg/day of estradiol valerate orally (pills group) and 38 received 4.5 mg/day of estradiol hemihydrate transdermally (gel group). Endometrial thickness was measured using transvaginal ultrasound between the 7 and 10th day of the cycle. Serum estradiol concentrations were measured on the day of initiating the cycle, on control transvaginal ultrasounds, and on the day of embryo transfer. Side effects were documented at each study visit. p<0.05 were adopted as statistically significant. The groups were compared using Student's t-test for continuous variables and chi-square or Fisher's exact test for categorical variables.

**RESULTS:**

There were no significant group differences (p>0.05) in endometrial thickness, biochemical and clinical pregnancy rates, miscarriage rate, blood estradiol concentrations, duration of estradiol administration, or cycle cancellation rates.

**CONCLUSION:**

Endometrial preparation with transdermal estrogen yielded similar reproductive outcomes to oral estrogen with fewer side effects.

## INTRODUCTION

Over the past decade, the proportion of frozen embryo transfers has increased substantially^1^. Despite its proven efficacy and indications, the ideal protocol for endometrial preparation (EP) for thawed embryo transfer remains the subject of debate^2–8^.

When transdermal estrogen is used, the metabolism of the first passage through the liver does not take place, with consequent lower stimulation of hepatic proteins and coagulation factors, and a neutral metabolic profile, which is potentially more favorable in terms of cardiovascular risk and thromboembolic events^9–12^.

In this context, the results of studies comparing the efficacy of different routes for estrogen in freeze–thaw embryo transfer cycles have been conflicting^8,13–16^.

The lack of investigations on this topic prompted the present study comparing two regimens of hormone replacement for EP in thawed embryo transfer: estrogen gel and estrogen pills.

## METHODS

We conducted a prospective randomized clinical trial of women undergoing treatment for cryopreserved embryo transfer to compare two EP protocols: estrogen gel and estrogen pills.

This was a single-center study conducted at a private reproductive center between June 2020 and October 2021. The study was approved by the Ethics Committee for Research in Humans of the Santa Casa of Sao Paulo School of Medical Science (Process number 23023219.2.0000.5479) in accordance with good clinical practice guidelines. Patients signed the informed consent form agreeing with the assisted reproduction treatment procedures according to local ethics regulations. It was also registered at the Registro Brasileiro de Ensaios Clínicos (ReBEC—Brazilian Clinical Trials Registry) under UTN (Universal Trial Number): A36950145802.

The study's inclusion criteria were as follows: women aged ≥21 and ≤38 years when undergoing embryo transfer using their own embryos, or those aged ≥21 and ≤50 years when undergoing embryo transfer using egg or embryo donation; women who had a body mass index (BMI) ≥18 and ≤35 kg/m^2^; and women who had one or two cryopreserved embryos from the 5 or 6th day.

However, patients were excluded from the study if one or more of the following were present: women with serum progesterone concentrations ≥1 ng/mL on the day of study commencement; a history of thrombosis; abnormal liver function; anatomical endometrial abnormality; BMI<18 and >35 kg/m^2^; and history of gastroplasty.

### Study protocol

Screening: Anamnesis, physical examination, and specific complementary tests were performed for high-complexity assisted reproduction, according to Brazilian Health Regulatory Agency (ANVISA).

Timepoint 0 (T0): After a basal ultrasonography, analysis of laboratory tests, application of the inclusion and exclusion criteria, and signing of the Free and Informed Consent Form, treatment was started on the 2nd and 3rd day of menstruation. At this initial stage, blood levels of progesterone, estradiol, ALT, and AST were determined and participants randomly allocated into the two study intervention groups at a ratio of 1:1 using randomly generated numbers (http://www.randomization.com):

Gel group—Transdermal route: two 0.75 mg pumps of estradiol hemihydrate every 8 h (Oestrogel^®^ Besins Healthcare, Belgium); pills group—oral route: 2 mg of estradiol valerate every 8 h (Primogyna^®^ Bayer, Germany).

Timepoint 1 (T1): Transvaginal ultrasound scan was performed between the 7 and 10th day of EP. If the endometrial thickness was ≥7 mm, then embryo transfer was scheduled, and women started taking vaginal micronized progesterone 600 mg/day (Utrogestan^®^, Besins Healthcare, Belgium) for 5–6 days before transfer.

Timepoint 2 (T2): In cases where the endometrium failed to exhibit satisfactory thickness, the estrogen dose was increased to 6 or 8 mg/day in the Gel and Oral groups, respectively. Women whose endometrium failed to reach the thickness or pattern required, even after increasing estrogen dose, were excluded from the study.

Timepoint 3 (T3—FET): Embryo transfer was performed. Following completion of transfer, participants were asked to do a pregnancy test (beta-hCG quantitative) after 10 days.

Timepoint 4 (T4): At 4–5 weeks after embryo transfer, in the event of a positive pregnancy test, a transvaginal obstetric ultrasound scan was performed.

All adverse symptoms were recorded at each of the study visits.

Serum estradiol concentration was measured in all women at baseline, at control transvaginal ultrasounds, and on the day of embryo transfer. Progesterone level was analyzed at baseline and at control ultrasounds. Transaminase levels were determined at the start of treatment and on the day of embryo transfer. All tests were carried out by the same laboratory.

Chemical pregnancy was defined as the presence of beta-hCG ≥ 25 UI/L at 10 days after embryo transfer. Clinical pregnancy was determined as the presence of a gestational sac on transvaginal ultrasound at 4–5 weeks after embryo transfer. Miscarriage was defined as a nonviable intrauterine pregnancy prior to 20 weeks gestation.

### Statistical study

Student's t-test was employed for calculating sample size, adopting a 5% level of significance and a test power of 80%. Data was drawn from a pilot study, which found a mean endometrial thickness of 7.35 mm and a standard deviation (SD) of 1.0. Differences of 10% above the mean were allowed for, giving a sample size of 30 participants per group.

For descriptive analysis of data, clinical and demographic characteristics were expressed as mean and SD for continuous variables, and as frequencies and percentages for categorical variables.

Regarding statistical tests, Student's t-test was used for continuous variables and the chi-square or Fisher exact test for categorical variables.

All statistical analyses were performed using the SPSS 21 software (IBM software) and p-values of <0.05 were considered statistically significant.

## RESULTS

A total of 88 women were included in the study. Overall, 93.1% (82/88) completed the study protocol, comprising 38 women in the transdermal estrogen group (gel group) and 44 women in the oral estrogen group (pills group).

**Figure 1:**
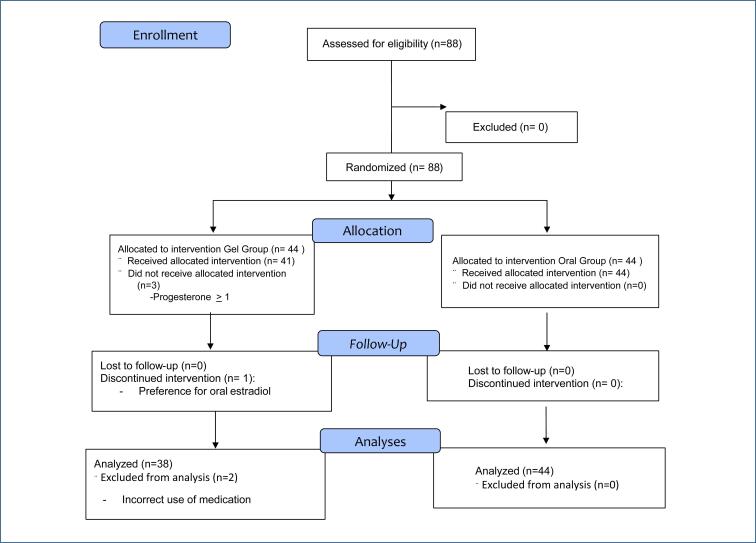
CONSORT flowchart of the trial.

Six women were excluded from the gel group: two for administering medication incorrectly; one for reporting difficulty applying the gel and deciding to take oral estrogen; and three for having progesterone level>1 ng/mL on day of treatment commencement. All participants in the pills group were included (Figure 1).

The gel and pills groups had similar characteristics for BMI, infertility time, egg/embryo donation, number of embryos transferred, and embryo quality (see Table 1).

**Table 1:** Basic and demographic characteristics of patients in two groups.

		Gel group	Pills group	p
		n=38	n=44	
		n (%)	n (%)	
Age (years)*		37.15±5.27◊	37.25±5.57◊	0.939
BMI (kg/m^2^)*		25.46±3.9◊	26.48±3.73◊	0.223
Duration of infertility (years)*		6.78±4.83◊	6.52±3.68◊	0.791
Cause of infertility**				
	Tuboperitoneal	14 (36.8%)	8 (18.1%)	0.057
	Ovulatory + male factor	3 (7.8%)	7 (15.9%)	0.326
	Male factor	6 (15.7%)	10 (22.7%)	0.429
	Unexplained	3 (7.8%)	4 (9.0%)	1
	Same sex couple/solo parent	5 (13.15%)	3 (6.8%)	0.462
	Tubal + male factor	1 (2.63%)	3 (6.8%)	0.620
	Ovulatory + male factor	4 (10.5%)	5 (11.3%)	1
	Tubal + ovulatory factor	2 (5.26%)	4 (9.0%)	0.679
Egg and embryo donation**				
	Own eggs	27 (71.1%)	30 (68.2%)	0.485
	Egg or embryo donation	11 (28.9%)	14 (31.8%)	
No. of embryos transferred**				
	1	10 (28.6%)	31 (73.8%)	0.815
	2	25 (71.4%)	11 (26.2)	
At least a top-quality embryo transferred**		6/35□ (17.1%)	6/42□ (14.3%)	0.977

N: no. of participants; %: percentage;

* Student's t-test;

** chi-square test;

◊ mean (±SD): standard deviation; p<0.05;

□ only cycles with embryo transfer.

The mean thickness of the endometrium thickness on the ultrasound performed between the 7 and 10th day of EP (T1) and was similar between both groups: 7.87±1.74 mm in the gel group versus 8.45±2.04 mm in the pills group (p=0.175) (Table 2—T1).

**Table 2:** Comparison of groups in relation to laboratory and ultrasound tests at T0, T1, T2, and frozen embryo transfer.

		Gel group	Pills group	p
		n=38	n=44	
		Mean±SD	Mean±SD	
T0	AST (U/L)*	17.57±4.10	21.58±9.20	0.016
	ALT (U/L)*	17.89±6.95	20.27±12.10	0.289
	P4 (ng/mL)*	0.25±0.15	0.33±0.25	0.086
	E2 (pg/mL)*	41.28±32.82	42.90±21.31	0.793
T1	ET (mm)*	7.87±1.74	8.45±2.04	0.175
	P4 (pg/mL)*	0.22±0.21	0.25±0.16	0.569
	E2 (ng/mL)*	275.79±193.40	224.57±83.24	0.116
	Endometrium trilinear**	32/38 (84.2%)◊	42/44 (95.5%)◊	0.087
	Adverse effects***	4 (10.5%)◊	15 (34.1%)◊	0.017
	Headache***	1 (2.6%)◊	8 (18.2%)◊	0.033
	Gastrointestinal***	0	5 (11.4%)◊	0.058
	Cramps***	1 (2.6%)◊	1 (2.3%)◊	1
	Paresthesia***	1 (2.6%)◊	1 (2.3%)◊	1
	Others***	1 (2.6%)◊	3 (6.8%)◊	0.365
		n=12	n=8	
T2	ET (mm)*	7.68±1.79	7.35±1.00	0.64
	P4 (ng/mL)*	0.15±0.11	0.19±0.27	0.722
	E2 (pg/mL)*	327.94±161.22	302.33±99.72	0.736
	EE trilinear***	10 (83.3%)◊	8 (100.0%)◊	0.224
		n=35	n=42	
FET	AST (U/L)*	25.36±13.05	28.15±21.16	0.593
	ALT (U/L)*	22.21±13.68	19.03±9.62	0.264
	E2 (pg/mL)*	226.24±138.44	224.35±91.50	0.945

N: no. of participants; SD: standard deviation; P4: progesterone; E2: estradiol; %: percentage; ET: endometrial thickness;

◊ n (%). T0: Initial time. T1: visit 1 of the study performed between the 7 and 10th day of endometrial preparation. T2: visit 2 of the study. FET: frozen embryo transfer.

* Student's t-test;

** chi-square test;

*** Fisher's exact test; p<0.05.

There were no significant group differences on laboratory tests performed 7–10 days into EP (Table 2).

Adverse effects were probed at 7–10 days of medication use. Women using oral estrogen (pills group) reported a higher rate of adverse effects compared to women using transdermal estrogen (gel group) (34.1 vs. 10.5%, p=0.0017). The most frequent adverse symptom in the pills group was headache, occurring in 18.2% of cases versus 2.6% in the gel group (p=0.033) (Table 2—T1).

Of the 82 cycles initially selected for the study, 20 women had endometrium thickness<7.0 mm after 7–10 days of estrogen use in both groups. As per the study protocol, estrogen dose was increased in these women and follow-up assessment performed after 5 days. No significant group difference in endometrial thickness/aspect or serum progesterone and estradiol concentration was evident (Table 2—T2).

After increasing the estrogen dose, 15 women attained endometrial thickness ≥7.0 mm and 5 had treatment cancellation due to insufficient endometrial thickness for embryo transfer. Of these cancellations, three were from the gel group and two from the pills group.

On the day of embryo transfer, the laboratory test results of the two intervention groups were compared. No significant group differences were detected for TGO, TGP, or estradiol (Table 2—FET).

Comparison of clinical outcomes showed no significant group differences, with results proving similar irrespective of route of estrogen administration (Table 3).

**Table 3:** Comparison of clinical outcomes of study groups.

	Gel group	Pills group	p
	n=38	n=44	
	n (%)	n (%)	
Duration of endometrial preparation (days)*	14.86±2.37◊	14.43±1,56◊	0.344
Cycle cancellation rate**	3/38 (7.9%)	2/44 (4.5%)	0.527
Biochemical pregnancy**	18/35□ (51.4%)	28/42□ (66.7%)	0.261
Clinical pregnancy**	11/35□ (31.4%)	16/42□ (38.1%)	0.542
Multiple pregnancy**	3/11Δ (27.3%)	3/16Δ (18.8%)	0.601
Miscarriage rate**	7/18 (38.8%)	12/28 (42.8%)	0.070
Live births per cycle transferred**	11 /35□ (31.4%)	16/42□ (38.1%)	0.542

N: no. of participants; %: percentage;

◊ Mean (±SD): standard deviation;

□ only cycles with embryo transfer,

Δ positive clinical pregnancy only.

* Student's t-test;

** chi-square test; p<0.05.

Regarding pregnancy rates, chemical pregnancy was 51.4 versus 66.7% (p=0.261) and clinical pregnancy 31.4 versus 38.1% (p=0.476) in the gel and pills groups, respectively.

Similarly, there were no significant differences between the gel and pills groups for abortion rates (38.8 vs. 42.8%) (p=0.070), live births per cycle transferred (25.7 vs. 38.09%) (p=0.275), duration of EP (14.86±2.37 vs. 14.43±1.56 days) (p=0.344), or cycle cancellation rate (7.9 vs. 4.5%) (p=0.527).

## DISCUSSION

The aim of the present study was to further the knowledge on hormone replacement regimens for EP in frozen–thawed embryo transfer.

Unlike most previous investigations assessing transdermal estrogen patches, the present study administered transdermal estrogen through the use of gel^15,17^.

The results of the present study on endometrial thickness measured at 7–-10 days of EP revealed no significant group difference between oral estrogen (pills group) and transdermal estrogen (gel group).

These findings corroborate the studies by Tehraninejad et al.^15^ and Garimella et al.^17^. These studies found no difference in endometrial thickness between the oral and transdermal estrogen groups.

In contrast to the present study and other investigations cited, Ferrer-Molina et al.^14^, in a prospective randomized study, found greater endometrial thickness in the transdermal estrogen (estradiol hemihydrate patches) group compared with the oral estrogen (estradiol valerate) group, with no apparent repercussions on the rate of pregnancy, miscarriages, or live births.

Thus, no significant group differences in pregnancy, miscarriage, or live birth rates were evident. These results are similar to those reported by Davar et al.^13^, whose prospective randomized study compared EP using transdermal estradiol 17-B patches against oral estradiol valerate, and also reflect the findings of Garimella et al.^17^.

Conversely, Tehraninejad et al.^15^ found a lower rate of miscarriage and a higher rate of ongoing pregnancy and live births in the group using transdermal estrogen gel than the group taking oral estrogen in pill form. According to the opinion of the authors, these effects might be explained by the high concentration of estradiol in the orally administered group or by the more physiological fluctuation in estrogen level associated with the transdermal route compared to oral administration. However, the authors emphasized the need for further trials to confirm their results.

For blood estradiol levels, no difference between the present study intervention groups was found at 7–10 days of EP. However, Garimella et al.^17^ and Tehraninejad et al.^15^ found significantly higher estradiol levels in women undergoing EP using oral estrogen pills than those who administered transdermal gel. The disparate result of the present study, differing from the findings of most of the cited publications, does not affect the rates of pregnancy, miscarriage, or live births.

Similar to the studies by Garimella et al.^17^ and Tehraninejad et al.^15^, no difference in EP cycle cancellation rate was found between the intervention groups.

Regarding adverse effects, rates were lower for the transdermal route (gel group) (10.5%) than the oral route (pills group) (34.1%) (p=0.017), with the most reported symptom of headache occurring in 2.6% versus 18.2%, respectively (p=0.033). Corroborating the present findings, Garimella et al.^17^ reported that a higher number of women had side effects in the oral group than gel group, where the most frequent adverse reaction reported in the oral group was gastrointestinal effects (30 vs. 1.4% p<0.01) and headache (17.3 vs. 3.6% p<0.01).

Contradicting these results, Ferrer-Molina et al.^14^ found that oral treatment was perceived as more comfortable than transdermal, a finding attributed to the high humidity of the city in which the study was carried out, claiming that many women had complaints regarding detachment of patches and skin reactions at the site of application.

Thus, the present study makes several contributions to clinical practice, with the absence of significant differences in endometrial thickness, rates of live births, pregnancy, or miscarriage between the intervention groups demonstrating similar efficacy. Moreover, the administration of estrogen gel has the added benefit of a lower rate of side effects. This study has special relevance in that there is a dearth of studies in the literature comparing the efficacy of estrogen gel versus oral estrogen in FET cycles.

Nonetheless, this study has some limitations. The analysis of the results was originally performed to consider endometrial thickness, but the rate of live births may be of greater clinical interest.

Finally, the results of this study are strengthened by its selection of the appropriate sample size for the primary objective and by the fact that both groups were homogeneous for baseline clinical and laboratory characteristics. Therefore, taken together, these results suggest that EP using estrogen gel can be offered as a first line for EP once it is associated with fewer side effects and has similar reproduction outcomes compared with oral estrogen.
